# The EU Health Task Force for emergency preparedness and response: overview of its first 2 years of operation, 2023 to 2025

**DOI:** 10.2807/1560-7917.ES.2026.31.14.2500753

**Published:** 2026-04-09

**Authors:** Orla Condell, Despina Pampaka, Adriana Romani, Daniel Cauchi, Dorothée Obach, Emma Löf, Ettore Severi, Stefania De Angelis, Adela Paez Jimenez, Alexandre Jully, Ana Ascenção e Silva, Andras Armväärt, Anna Battistutta, Bernardo Guzman-Herrador, Claudia Siffczyk, Daniela Garone, Dimitrios Paraskevis, Emily MacDonald, Evelyn Depoortere, Gianluca Loi, Guido Benedetti, Isabella Panunzi, Jorgen Stassijns, Laura Gillini, Máirín Boland, Manuela Mura, Oleg Storozhenko, Otto Helve, Panu Saaristo, Paula Vasconcelos, Ricardo Mexia, Sébastien Français, Thomas Hoffman, Vicky Lefevre

**Affiliations:** 1 European Centre for Disease Prevention and Control (ECDC), Stockholm, Sweden; 2 EU Health Task Force External Advisory Group, Stockholm, Sweden; 3 World Health Organization (WHO) Regional Office for Europe, Copenhagen, Denmark; 4 EU Health Task Force Ad Hoc Working Group, Stockholm, Sweden; 5 European Commission, Brussels, Belgium; 6 Public health emergencies preparedness department, Health Board, Tallinn, Estonia; 7 Coordinating Centre for Health Alerts and Emergencies (CCAES), Ministry of Health, Madrid, Spain; 8 Unit for crisis management, outbreak investigations & training programmes, Department of Infectious Disease Epidemiology, Robert Koch-Institute, Berlin, Germany; 9 Médecins Sans Frontières International, Geneva, Switzerland; 10 National Public Health Organization, Athens, Greece; 11 Norwegian Institute of Public Health, Oslo, Norway; 12 Global Outbreak Alert and Response Network, WHO Health Emergencies Programme, World Health Organization, Geneva, Switzerland; 13 Department of Infectious Disease Epidemiology and Prevention, Statens Serum Institut, Copenhagen, Denmark; 14 Crisis Preparedness and Response, Sciensano, Brussels, Belgium; 15 European Commission, Luxembourg, Luxembourg; 16 Department of Public Health, HSE Dublin and Midlands, Dublin, Ireland; 17 Department of Public Health Threats, European Medicines Agency (EMA), Amsterdam, Netherlands; 18 Department of Public Health, Finnish Institute for Health and Welfare, Helsinki, Finland; 19 International Federation of Red Cross and Red Crescent Societies, Europe Region, Budapest, Hungary; 20 Public Health Emergencies Operations Centre, Directorate-General of Health, Lisbon, Portugal; 21 European Public Health Association (EUPHA), Utrecht, Netherlands; 22 Emergency Preparedness and Response Unit, Health Directorate, Ministry of Health and Social Security, Luxembourg, Luxembourg

**Keywords:** European Union, Emergency response, Preparedness, Outbreaks and crises, Deployments

## Abstract

The European Union (EU) Regulation on serious cross-border threats to health (2022/2371) and ECDC’s enhanced mandate (2022/2370) established the EU Health Task Force (EUHTF) as a deployable public health workforce providing emergency response and preparedness support globally during EU Public Health Emergency and non-emergency periods. The EUHTF comprises an ECDC Coordination Team and three expert pools: the ECDC expert pool, the ECDC fellowship pool and the external expert pool. The EUHTF’s establishment and operationalisation was guided by an advisory group with representatives from EU/EEA countries, the European Commission, the European Medicines Agency, the World Health Organization Global Outbreak Alert and Response Network and other international stakeholders. Mechanisms were developed for EUHTF logistics within the EU/EEA, while formalised partnerships facilitate operations beyond the EU/EEA. From its inception in January 2023 to 31 December 2025, the EUHTF supported 31 requests: 23 from 15 EU/EEA countries, including 19 on preparedness and four on outbreak response, and eight emergency response assignments from outside the EU/EEA. The EUHTF facilitates stronger EU/EEA-level support for disease outbreaks inside and outside the EU/EEA, enhances ECDC’s capacity to mobilise European expertise and enables close collaboration with global partners and EU/EEA countries to ensure coordination and efficient use of resources.

## Background

The COVID-19 pandemic revealed shortcomings in global preparedness for, and in response to, public health threats, such as a deployable public health workforce. To overcome this challenge, in May 2023 the World Health Organization (WHO) launched the Global Health Emergency Corps (GHEC) to strengthen worldwide capacity for rapid response to health crises [1]. At the European Union (EU) level, the COVID-19 pandemic highlighted that preparedness plans and capacities across EU/European Economic Area (EEA) countries and EU institutions were insufficient to meet the challenges of the pandemic, whereas existing EU response frameworks lacked adequate resources, coordination and capabilities for deployment of rapid response and surge capacities [2-4]. These lessons from the COVID-19 pandemic and lessons from other public health crises underscore the need for a strengthened health emergency workforce, international coordination for crisis preparedness and enhanced, agile mechanisms to respond to public health emergencies at a regional and country level [3,5]. Consequently, in November 2022, the EU adopted a comprehensive legislative package, extending the European Centre for Disease Prevention and Control’s (ECDC) mandate and establishing the EU Health Task Force (EUHTF) [6-8]. This perspective describes the establishment of the EUHTF and its first 2 years of operation.

## The establishment of the EU Health Task Force

The EUHTF was established in January 2023 as a deployable public health workforce that offers emergency preparedness and crisis response support related to communicable diseases and diseases of unknown origin [8], enhancing ECDC’s capacity to mobilise tailored technical expertise. The EUHTF aims to ensure efficient resource utilisation and avoid duplication by collaborating with global partners, including the European Commission’s Directorate-General for European Civil Protection and Humanitarian Aid Operations (DG ECHO) and the WHO Global Outbreak Alert and Response Network (GOARN), among others.

## Composition, support areas and operational procedures

The EUHTF comprises a permanent ECDC Coordination Team and three expert pools. The ECDC Coordination Team establishes the EUHTF working modalities, objectives, tasks and expert pools, oversees daily operations, coordinates assignments and engages in technical work.

The three EUHTF expert pools provide expertise to fulfil EUHTF assignments and comprise: (i) the ECDC expert pool of ECDC technical staff, (ii) the ECDC fellowship pool of fellows enrolled in ECDC fellowship or associated programmes [9], and (iii) the external expert pool of technical experts who are EU/EEA nationals or third-country nationals working in the EU/EEA. The latter pool is being established following an iterative approach and is currently restricted to ECDC fellowship alumni before further expansion.

For specific EUHTF assignments, expert pool members are invited to express their interest to contribute and are selected based on technical competencies, qualifications, experience, availability and other criteria. To make the pool attractive and increase readiness for rapid enrolment of trained experts into EUHTF assignments, expert pool members are invited to join a community of practice and are offered technical training opportunities (e.g. training on simulation exercises, outbreak investigations and the use of outbreak investigation tools).

Requests for EUHTF support should focus on technical areas across the entire preparedness and response cycle, from reviewing national prevention, preparedness and response (PPR) plans to supporting emergency response or outbreak investigation (Table 1) including monitoring of mass gatherings, capacity building and simulation exercises. Assignments, which must have a clear expected public health impact and can tackle broad or specific areas, may vary in duration but are always time limited (e.g. 6 weeks for exclusive/full-time work, or longer for non-exclusive/part-time work). The EUHTF works globally but serves, in order of priority, EU/EEA countries, EU accession countries, potential EU candidate countries and European Neighbourhood Policy countries [10]. In addition, the EUHTF gives priority to requests for response activities over preparedness work, especially during a public health crisis and particularly for events with a potential for multi-country spread or those with a high severity and/or high public health impact. If a public health emergency is declared at EU level, the EUHTF Enhanced Emergency Capacity can be activated upon request from two EU/EEA countries and the European Commission. The Enhanced Emergency Capacity engages all EUHTF resources and is coordinated jointly by ECDC and the Health Security Committee hosted by the European Commission’s Directorate-General for Health and Food Safety (DG SANTE) to ensure transparency, information sharing and inputs from all EU/EEA countries [7,11].

**Table 1:** Technical areas and potential activities for EU Health Task Force assignments

Areas for EUHTF assignments	Examples
**Preparedness**	
Developing, reviewing of preparedness and response plans or guidelines/protocols related to specific technical areas	Development/review of needs-assessments, plans and protocols for specific technical areas e.g. surveillance, public health microbiology, infection prevention and control, emergency response operations, border-health, One Health
Testing of preparedness and response plans, or plans related to specific technical areas or diseases	Simulation exercises
Reviewing implementation of preparedness and response actions	In-action reviews, after-action reviews
Supporting implementation of capacity-building activities	Support implementation of specific training needs, such as those identified during public health emergency preparedness assessments (PHEPA)
**Response**	
Threat detection and monitoring, including in advance of mass gathering events or during outbreaks or following disasters	Epidemic intelligence activities, developing, strengthening or operating surveillance systems
Outbreak investigation and emergency response	Epidemiological investigations, including for a new/emerging or rare pathogens, rapid threat and risk assessments, molecular epidemiology, risk communication, recommendations for preparedness and response options, response support to humanitarian crises / natural disasters with infectious disease risk
Emergency operations centre tools or procedures	Support the development and implementation of an incident management system and/or development and maintenance of incident management tool(s)
Operational research for evidence gathering in the context of an outbreak	Developing protocols, supporting cross border studies including data integration and analysis, supporting collaboration with external partners/stakeholder including relevant EU initiatives, etc.

EU: European Union; EUHTF: European Union Health Task Force; PHEPA: public health emergency preparedness assessments.

## Collaborations and partnerships

For operations within the EU/EEA, the EUHTF manages logistics and costs directly. Operations beyond the EU/EEA are generally conducted in collaboration with GOARN and/or DG ECHO.

The WHO Global Outbreak Alert and Response Network mobilises technical partners from its global network to support response to public health emergencies. The EUHTF assists with mobilising EUHTF experts and EU funding in response to GOARN requests for assistance such as deployments to support WHO and other United Nations organisations. The European Centre for Disease Prevention and Control is both a GOARN partner and part of the GOARN Steering Committee. This ensures coordination and complementarity between the EUHTF and GOARN’s global response mechanisms.

Under formal agreements with DG ECHO, the EUHTF can deploy experts during public health crises with humanitarian dimensions through DG ECHO’s deployment and funding mechanisms, including ReliefEU, the Union Civil Protection Mechanism (UCPM) and rescEU [12,13]. The collaboration builds on previous experiences, such as that of the European Medical Corps (EMC) [14] and is of mutual benefit, extending DG ECHO’s focus to deployable response capacity for infectious disease events.

Through DG ECHO and GOARN, the EUHTF may deploy experts within WHO’s Emergency Medical Team (EMT) and the Rapid Response Mobile Laboratories network [15,16], reinforcing the effectiveness of crisis response through coordinated public health expertise and action.

In practice, when the EUHTF provides support in collaboration with another regional or global initiative, it aims to integrate its experts into the operational teams that requested support in the field. Leadership and direction for the assignment are provided by the operational teams, with the EUHTF Coordination Team taking a supporting or advisory role, as necessary.

Similarly, when the scope of requests to the EUHTF overlaps with the roles of other European Commission bodies or European agencies such as the European Medicines Agency (EMA), the European Commission’s Directorate-General for Health Emergency Preparedness and Response Authority (DG HERA) or other disease control centres such as Africa Centres for Disease Control and Prevention (Africa CDC), the EUHTF follows a collaborative approach, working with counterparts as necessary and appropriate. In our opinion, the EUHTF response to the mpox outbreak in the Democratic Republic of Congo in 2024 illustrates its collaborative approach, ensuring complementarity among response mechanisms, avoiding duplication of efforts and promoting efficient resource use.

## Governance

In addition to standard ECDC organisational rules and governance bodies, the EUHTF has established governance structures to ensure interactions and facilitate cooperation with EU/EEA countries and international partners. In 2023, the EUHTF ad hoc working group was established with representatives from six EU/EEA countries, four European Commission Directorates-General (DG ECHO, DG HERA, the Directorate-General for Research and Innovation (DG RTD) and DG SANTE) and GOARN (Figure). This working group advised the ECDC Coordination Team on defining the EUHTF’s initial arrangements, processes and functioning.

**Figure:**
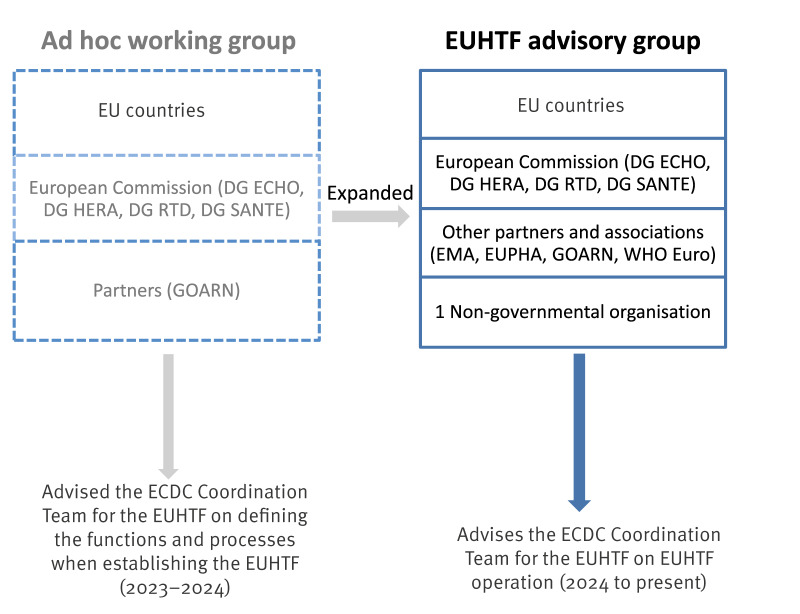
Structure and role of advisory groups in the governance of the EU Health Task Force

DG ECHO: the European Commission’s Directorate-General for European Civil Protection and Humanitarian Aid Operations; DG HERA: the European Commission’s Directorate-General for Health Emergency Preparedness and Response Authority; DG RTD: the European Commission’s Directorate-General for Research and Innovation; DG SANTE: the European Commission’s Directorate-General for Health and Food Safety; ECDC: European Centre for Disease Prevention and Control; EMA: European Medicines Agency; EU: European Union; EUHTF: European Union Health Task Force; EUPHA: European Public Health Association; GOARN: World Health Organization Global Outbreak Alert and Response Network; WHO Euro: World Health Organization Regional Office for Europe.

In 2024, the EUHTF ad hoc working group was replaced by the EUHTF advisory group, which continues to provide support and advice on operational, administrative and technical decisions. It includes additional representatives from EMA, the WHO Regional Office for Europe, the European Public Health Association and a non-profit humanitarian non-governmental organisation (NGO). Representatives from EU/EEA countries and NGO members rotate every 2 years, providing opportunities to participate in the advisory group and bring diverse perspectives and experience (Figure).

## EU Health Task Force assignments as of December 2025

Between January 2023 and December 2025, the EUHTF received 36 activation requests. Three were not accepted. Of these, two aligned with programmatic work related to epidemic intelligence or the European surveillance portal for infectious diseases (EpiPulse) support and were supported by ECDC outside the EUHTF framework, while one related to environmental testing standards and therefore fell outside the ECDC mandate. Of the 33 accepted, two were later discontinued by the requesting countries. The remaining 31 assignments included 23 in EU/EEA countries and eight outside the EU/EEA (Table 2). Most (19/23) EU/EEA assignments focused on preparedness, while four related to outbreak response. All assignments carried out outside the EU/EEA were related to emergency response.

**Table 2:** Summary of all EU Health Task Force assignments (completed and ongoing), 2023–2025

Year started	Country	Technical area/Assignment topic	Location/means support was provided		Duration (weeks)^a^
			Remote	In-person	
**Requests within EU/EEA countries**					
**Preparedness**					
2023	Lithuania	AAR: contact tracing during the COVID-19 pandemic	Yes, preparing for the AAR	Yes, delivering the AAR workshop	20
2023	Slovenia	AAR: response measures, vaccination and risk communication during the COVID-19 pandemic	Yes, preparing for the AAR and writing an AAR report [18]	Yes, delivering the AAR workshop	38
2023	Latvia	Training on surveillance, data collection and outbreak investigation	Yes, preparing the material for the training	Yes, delivering the training	26
2024	France	Epidemic intelligence and response for two mass gathering events	Yes, monitoring by the Epidemic Intelligence team	No	9
2024	Germany	Risk assessment in advance of and monitoring during a mass gathering event	Yes	No	26
2024	Germany	AAR: coordination between public health and international airport authorities during the COVID-19 pandemic	Yes, preparation for the AAR and drafting the final AAR report	Yes, delivering the AAR workshop	23
2024	Portugal	AAR: testing and contract tracing during the COVID-19 pandemic	Yes, preparing for the AAR	No	3
2023	Slovenia	Guidance on national workforce capacity estimation	Yes	Yes, on-site visit	1
2025	Malta	CBRN threat training	No (only administrative remote support)	Yes, participation in ECDC-arranged CBRN training	< 1
2025	Malta	Development of a national PPR plan	Yes, organising a webinar	No	Ongoing
2025	Lichtenstein	Development of a national PPR plan	Yes, organising a webinar	No	Ongoing
2025	Cyprus	Development of a national PPR plan	Yes, organising a webinar	No	Ongoing
2025	Greece	Expert advice on capacity building for surveillance, preparedness and response	No	Yes, on-site visit	< 1
2025	Poland	Development of an EOC	Yes, online meetings	Not yet	Ongoing
2025	Malta	Designing and delivering a SimEx	Yes, preparing for the SimEx (ongoing)	Yes, delivering the SimEx	Ongoing
2025	Liechtenstein	Designing and delivering a SimEx	Yes, preparing for the SimEx (ongoing)	Yes, delivering the SimEx (planned)	Ongoing
2025	Luxembourg	Advice on development of a National Action Plan for Health Security (post-PHEPA)	Yes, preparing for the meeting	Yes, on-site meeting with experts	
2025	Belgium	Designing and delivering a SimEx	Yes, preparing for the SimEx (ongoing)	Yes, delivering the SimEx (planned)	Ongoing
2025	Slovenia	Development of an EOC	Yes, consultation was provided on several occasions, in advance of the site-visit	Yes, on-site visit at ECDC	11
**Response**					
2023	Ireland, multi-country	Multi-country operational research: invasive group A streptococcal infection risk factors and surveillance [19]	Yes, online meetings and support from the conception of the research work until submission of the manuscript	No	86
2024	Romania	Measles data management support	Yes	No	32
2025	Latvia	Support outbreak investigation of Shiga toxin-producing *Escherichia coli*	Yes, remote support was provided by the data scientists on duty and by the experts deployed post-mission	On-site deployment	3
2025	Latvia	Expert advice on rodent control during a leptospirosis outbreak	Yes, online meetings and sharing relevant technical material	No	3
**Requests outside EU/EEA countries**					
**Response**					
2023	Ukraine	RRA related to a flooding event	Yes	No	1
2024	Jordan	Response to infectious disease events associated with the escalation of violence in Israel and occupied Palestinian territories, through GOARN and DG ECHO (based at UNRWA, in Jordan)	No	Yes, on-site deployment	12
2024	Zambia	Response to cholera epidemic in Zambia, through GOARN and DG INTPA	No	Yes, on-site deployment	4
2024	DRC	Response to mpox outbreak in the DRC, through GOARN, DG ECHO and DG INTPA	No	Yes, on-site deployment	21
2024	DRC	Response to the mpox outbreak in DRC, data analysis	Yes	No	10
2024	Rwanda	Response to Marburg virus disease outbreak in Rwanda, through DG ECHO via the UCPM	No	Yes, on-site deployment	2
2025	Sierra Leone	Response to the mpox outbreak through DG ECHO via the UCPM	Yes, some post-deployment remote support	Yes, on-site deployment	3
2025	DRC	Response to the Ebola virus disease outbreak through DG INTPA. Joint deployment with Africa CDC	No	Yes, on-site deployment for	3

AAR: after-action review; CBRN: chemical, biological, radiological and nuclear; CDC: Centers for Disease Control and Prevention; DG ECHO: the European Commission’s Directorate-General for European Civil Protection and Humanitarian Aid Operations; DG INTPA: the European Commission’s Directorate-General for International Partnerships; DRC: Democratic Republic of Congo; ECDC: European Centre for Disease Prevention and Control; EOC: emergency operations centre; GOARN: World Health Organization Global Outbreak Alert and Response Network; PHEPA: public health emergency preparedness assessment; PPR: prevention, preparedness and response; RRA: rapid risk assessment; SimEx: simulation exercise; UCPM: the Union Civil Protection Mechanism; UNRWA: United Nations Relief and Works Agency for Palestine Refugees in the Near East.

^a^ The duration of preparedness assignments is measured from the initiation of activities to their completion and generally reflects part-time engagement, which varies depending on the topic. For response assignments, the duration corresponds to the weeks spent in the field; when remote support continues after an on-site mission, the assignment concludes once this post-mission remote work is completed.

The preparedness activities included development of national PPR plans, after-action reviews, simulation exercises, capacity building and development of emergency operations centres. The assignments outside the EU/EEA related to emergency or outbreak response, including expert-deployments to the Democratic Republic of the Congo, Jordan, Rwanda, Sierra Leone and Zambia.

Fifty experts were engaged to fulfil these assignments: 40 from the ECDC expert pool, five from the ECDC fellowship pool and five from the EUHTF external expert pool. Thirteen experts from the ECDC expert pool contributed to more than one assignment. Overall, 17 experts were deployed on-site for the duration of their assignment, while 44 (including some who were also deployed internationally) supported remotely or through short on-site missions.

As at 31 December 2025, all assignments inside the EU/EEA have been funded and supported by ECDC. Assignments outside the EU/EEA bear higher costs and logistics and security challenges. Therefore, to use appropriate funding mechanisms and maximise deployment safety, three assignments were fully or partly implemented in collaboration with GOARN with costs borne by the EUHTF, DG ECHO and European Commission’s Directorate-General for International Partnerships (DG INTPA), while two assignments were conducted solely in collaboration with DG ECHO through UCPM funding (Table 2).

## Lessons learned, future direction

The EUHTF’s flexible set-up, offering in-person, remote or combined technical assistance, has responded to diverse requests. It will continue to offer tailored solutions so that countries and international organisations can strengthen best practices, be consistent in emergency preparedness and response and facilitate the exchange of experience and skills.

Within the EU/EEA, beyond specific national assignments, the EUHTF will continue to focus on enhancing cross-border preparedness and response collaboration during, for instance, mass gatherings and large outbreaks. Collaborating with the EUHTF has the potential to benefit requesting parties as well as assigned experts and has a positive impact in EU and global Health Security. Countries may receive rapid and tailored support, often at no cost. Improved national and cross-border preparedness and response capacity will enhance EU-wide resilience, whereas experts in the EUHTF pools may gain technical experience, build networks and contribute to knowledge exchange for their EU/EEA country.

To prepare for large-scale mobilisation during EU-declared public health emergencies, the EUHTF will expand its external expert pool. A versatile group of technical experts underpins the agility of the EUHTF, which draws upon ECDC staff’s technical expertise and management skills and ECDC fellows’ dynamism and analytical public health skills, as well as diverse language abilities enabling support to EU/EEA countries in their native language. The EUHTF external expert pool introduces a new approach to engaging EU/EEA experts in ECDC activities and will expand iteratively allowing access to strong expertise, not only in EU/EEA public health institutes and health ministries, but also potentially in academia and NGOs.

The close engagement between the EUHTF ad hoc working group and EUHTF advisory group has been instrumental in shaping the EUHTF’s development and operations while reflecting EU/EEA countries’ perspectives on gaps and added value of the task force. Similarly, collaborations with partner agencies and the Commission services facilitated effective integration of the EUHTF within the EU/EEA and the global health landscape. The EUHTF will continue close engagement with the EUHTF advisory group to align with EU/EEA countries’ and partners’ views. Moreover, the EUHTF will engage with EU/EEA countries’ focal points to ensure that countries stay informed of developments and liaise closely with the EUHTF.

Furthermore, the EUHTF will synergise with partners to respond to infectious disease events outside the EU/EEA. Ongoing collaboration with GOARN will be strengthened by franchising the GOARN Outbreak Response Scenario Training [17], enabling EUHTF expert pools to train to be part of an international outbreak response team, preparing them for GOARN deployments and field response. The EUHTF will continue collaborating with DG ECHO to assist in disaster and humanitarian crisis response and with DG SANTE and DG HERA to share expertise when requests and assignments may benefit from it, so as to increase deployment agility instead of duplicating partners’ support arrangements.

There are some limitations to this perspective. The primary aim is to provide a descriptive overview of the EU Health Task Force processes, rather than to describe and evaluate individual assignments in detail. An evaluation will be conducted in the future once a greater number of assignments has been completed. We therefore did not assess the acceptability of the EUHTF among stakeholders, nor did we assess the timeliness of the assignments, or how effectively they met their objectives.

## Conclusions

As a new initiative whose effectiveness depends on the engagement and utilisation of the different EU/EEA countries, raising awareness about the EUHTF’s scope and procedures, particularly within the EU/EEA, is essential. The EUHTF will continue to evolve, incorporating lessons learned to enhance operations and maximise its value to EU/EEA countries and global health security. The EUHTF will work to ensure that all EU/EEA countries and collaborating international organisations can count on its robust support capacities, independently from their varying preparedness and readiness levels.

## Data Availability

Data sharing is not applicable to this article as no datasets were generated or analysed during the current work.

## References

[1] World Health Organization (WHO). Global health emergency corps framework. Geneva: WHO; 2025. Available from: https://www.who.int/publications/b/78043#:~:text=The%20Global%20Health%20Emergency%20Corps,emergency%20preparedness%20and%20response%20architecture

[2] European Commission. Communication from the Commission to the European Parliament, the Council, the European Economic and Social Committee and the Committee of the Regions. Building a European Health Union: reinforcing the EU’s resilience for cross-border health threats. Brussels: European Commission; 2020. Available from: https://eur-lex.europa.eu/legal-content/EN/TXT/?uri=COM:2020:724:FIN

[3] European Commission. Communication from the Commission to the European Parliament, the Council, the European Economic and Social Committee and the Committee of the Regions. Drawing the early lessons from the COVID-19 pandemic. Brussels: European Commission; 2021. Available from: https://eur-lex.europa.eu/legal-content/EN/TXT/?uri=COM:2021:380:FIN

[4] Mauer N Panteli D Kahr-Gottlieb D De La Mata I. Towards a European Health Union: New instruments for stronger and more resilient health systems. Eurohealth (Lond). 2022;28(1). Available from: https://iris.who.int/server/api/core/bitstreams/b1ab17e4-1f3e-46e3-aa35-cffcbfbafddf/content

[5] Gontariuk M Krafft T Rehbock C Townend D Van der Auwermeulen L Pilot E. The European Union and Public Health Emergencies: Expert Opinions on the Management of the First Wave of the COVID-19 Pandemic and Suggestions for Future Emergencies. Front Public Health. 2021;9:698995. 10.3389/fpubh.2021.698995 34490183 PMC8417533

[6] European Parliament and Council of the European Union. Regulation (EU) 2022/2370 of the European Parliament and of the Council of 23 November 2022 amending Regulation (EC) No 851/2004 establishing a European centre for disease prevention and control. Official Journal of the European Union. 2022;L314:1-21. Available from: https://eur-lex.europa.eu/eli/reg/2022/2370/oj

[7] European Parliament and Council of the European Union. Regulation (EU) 2022/2371 of the European Parliament and of the Council of 23 November 2022 on serious cross-border threats to health and repealing Decision No 1082/2013/EU. Official Journal of the European Union. 2022;L314:26-63. Available from: https://eur-lex.europa.eu/eli/reg/2022/2371/oj

[8] Kokki M Ammon A. Preparing Europe for future health threats and crises - key elements of the European Centre for Disease Prevention and Control’s reinforced mandate. Euro Surveill. 2023;28(3):2300033. 10.2807/1560-7917.ES.2023.28.3.2300033 36695487 PMC9853949

[9] European Centre for Disease Prevention and Control (ECDC). Fellowship programme: EPIET/EUPHEM. Stockholm: ECDC. [Accessed: 26 Jun 2025]. Available from: https://www.ecdc.europa.eu/en/epiet-euphem#:~:text=The%20two-year%20ECDC%20Fellowship%20programme%20has%20two%20alternative,Programme%20fellows%20are%20placed%20in%20different%20training%20sites

[10] European Centre for Disease Prevention and Control (ECDC). EU Health Task Force. Stockholm: ECDC. [Accessed: 19 May 2025]. Available from: https://www.ecdc.europa.eu/en/about-ecdc/what-we-do/partners-and-networks/support-and-services-eueea-countries/health-task-force

[11] European Commission. Commission Implementing Regulation (EU) 2025/1536 of 29 July 2025 setting out the procedure concerning the mobilisation of the enhanced emergency capacity of the EU Health Task Force. Official Journal of the European Union. 2025;L1536. Available from: https://eur-lex.europa.eu/eli/reg_impl/2025/1536/oj

[12] European Commission. EU Civil Protection Mechanism. Brussels: European Commission. [Accessed: 19 May 2025]. Available from: https://civil-protection-humanitarian-aid.ec.europa.eu/what/civil-protection/eu-civil-protection-mechanism_en

[13] European Commission. European Humanitarian Response Capacity (EHRC). Brussels: European Commission. [Accessed: 19 May 2025]. Available from: https://civil-protection-humanitarian-aid.ec.europa.eu/what/humanitarian-aid/european-humanitarian-response-capacity-ehrc_en#:~:text=The%20European%20Humanitarian%20Response%20Capacity%20%28EHRC%29%20is%20a,response%20to%20sudden-onset%20natural%20hazards%20and%20human-induced%20disasters

[14] Haussig JM Severi E Baum JH Vanlerberghe V Laiseca A Defrance L The European Medical Corps: first Public Health Team mission and future perspectives. Euro Surveill. 2017;22(37):30613. 10.2807/1560-7917.ES.2017.22.37.30613 28933343 PMC5607656

[15] World Health Organization (WHO). Welcome to the WHO emergency medical teams initiative. Geneva: WHO. [Accessed: 16 March 2026]. Available from: https://extranet.who.int/emt/

[16] World Health Organization (WHO) Regional Office for Europe. Rapid Response Mobile Laboratories (‎RRML)‎ Network: at a glance. Copenhagen: WHO Euro; 2023. Available from: https://iris.who.int/handle/10665/365993

[17] Christensen R Fisher D Salmon S Drury P Effler P. Training for outbreak response through the Global Outbreak Alert and Response Network. BMC Med. 2021;19(1):123. 10.1186/s12916-021-01996-5 33985496 PMC8118749

[18] European Centre for Disease Prevention and Control (ECDC). After-Action review of the public health response of Slovenia to the COVID-19 pandemic. Stockholm: ECDC; 2023. Available from: https://www.ecdc.europa.eu/en/publications-data/covid-19-after-action-review-public-health-response-slovenia

[19] Cardoso MJ Obach D Löf E Marrone G Cornelissen L Charalambous M Multi-country surveillance of paediatric invasive group A Streptococcus infection, European Union/European Economic Area countries, 2022/23 season. Euro Surveill. 2025;30(42):2500079. 10.2807/1560-7917.ES.2025.30.42.2500079 41133308 PMC12555117

